# The Effect of Bismuth and Tin on Methane and Acetate Production in a Microbial Electrosynthesis Cell Fed with Carbon Dioxide

**DOI:** 10.3390/molecules29020462

**Published:** 2024-01-17

**Authors:** Rihab Gharbi, Sasha Omanovic, Sabahudin Hrapovic, Emmanuel Nwanebu, Boris Tartakovsky

**Affiliations:** 1 Department of Chemical Engineering, McGill University, 3610 University St., Montreal, QC H3A 0C5, Canada; 2 National Research Council of Canada, 6100 Royalmount Avenue, Montreal, QC H4P 2R2, Canada

**Keywords:** microbial electrosynthesis, transition metals, Bi, Sn, CO_2_ conversion, methane, acetate

## Abstract

This study investigates the impacts of bismuth and tin on the production of CH_4_ and volatile fatty acids in a microbial electrosynthesis cell with a continuous CO_2_ supply. First, the impact of several transition metal ions (Ni^2+^, Fe^2+^, Cu^2+^, Sn^2+^, Mn^2+^, MoO_4_^2−^, and Bi^3+^) on hydrogenotrophic and acetoclastic methanogenic microbial activity was evaluated in a series of batch bottle tests incubated with anaerobic sludge and a pre-defined concentration of dissolved transition metals. While Cu is considered a promising catalyst for the electrocatalytic conversion of CO_2_ to short chain fatty acids such as acetate, its presence as a Cu^2+^ ion was demonstrated to significantly inhibit the microbial production of CH_4_ and acetate. At the same time, CH_4_ production increased in the presence of Bi^3+^ (0.1 g L^−1^) and remained unchanged at the same concentration of Sn^2+^. Since Sn is of interest due to its catalytic properties in the electrochemical CO_2_ conversion, Bi and Sn were added to the cathode compartment of a laboratory-scale microbial electrosynthesis cell (MESC) to achieve an initial concentration of 0.1 g L^−1^. While an initial increase in CH_4_ (and acetate for Sn^2+^) production was observed after the first injection of the metal ions, after the second injection, CH_4_ production declined. Acetate accumulation was indicative of the reduced activity of acetoclastic methanogens, likely due to the high partial pressure of H_2_. The modification of a carbon-felt electrode by the electrodeposition of Sn metal on its surface prior to cathode inoculation with anaerobic sludge showed a doubling of CH_4_ production in the MESC and a lower concentration of acetate, while the electrodeposition of Bi resulted in a decreased CH_4_ production.

## 1. Introduction

Ever since the industrial revolution, fossil fuels, such as coal and petroleum, have become the main source of power production. The use of these resources produces greenhouse gases, which are pollutants that trap heat inside the atmosphere by absorbing the sunlight’s radiation through molecular bond vibrations. The increasing dependence on fossil fuels has resulted in global warming, with considerable consequences on the ecosystem and biodiversity of the Earth [1].

The most abundant greenhouse gas is carbon dioxide (CO_2_), which is estimated to contribute about 65% of the greenhouse gas effect [2]. Due to the consequences of global warming, CO_2_ capture, storage, and conversion have become of great interest. Converting CO_2_ to fuels is a way to avoid the long-term storage of captured CO_2_ and reduce our dependence on fossil fuels at the same time [3]. However, CO_2_ is a stable compound due to its molecular structure; it is, therefore, energetically difficult to chemically convert it to other molecules [4].

Research has shown many advancements in the electrochemical reduction of CO_2_, including the utilization of transition metals to selectively form certain products [5]. In particular, the recent study of Chen et al. [6] found that (Ir_1_)-doped hybrid Cu_3_N/Cu_2_O is a highly efficient catalyst for the conversion of CO_2_ to CH_4_. Additionally, Chen et al. [7] documented an in situ molecule modification strategy of a Cu-based cathode, effectively enhancing the selectivity of CO_2_ reduction to ethylene. This modification resulted in a 6.5 times improvement compared to an unmodified Cu cathode. In spite of these and other recent advancements, the electrocatalytic reduction of CO_2_ still faces such challenges as low selectivity, relatively low current density, high energy consumption, and insufficient catalyst stability [8].

The microbial electrosynthesis cell (MESC) is a type of bio-electrochemical system that has attracted research interest. In this system, the conversion of CO_2_ into value-added products is facilitated by microorganisms which are grown on the cathode [9,10]. Compared to the traditional electrochemical approach, an MESC is advantageous due to its low energy consumption, relatively stable rate of product formation, self-proliferating catalyst (microbial biofilm), and ability to operate under ambient conditions with a less intensive energy input [11,12]. This CO_2_ utilization technology is therefore economical and safe to operate. However, it is limited by its low volumetric conversion rates and low current density [13].

MESC performance is dependent on several factors, including the cathode material [14]. The cathode is highly significant as it acts as the site for microorganism growth, electron transfer, and electron uptake by the biofilm. Consequently, the cathode must have some essential characteristics. To ease electron transfer by the cathode and its uptake by the microorganisms, it is important that the cathode material has a high electrical conductivity. To ensure extensive biofilm formation, the cathode material must be non-toxic and have excellent biocompatibility. To improve the contact between microorganisms and the electrode and increase the volumetric biofilm density, a high electrode specific surface area is desired. In addition, to ensure the longevity of the MESC, the cathodic material must have a high corrosion resistance [4].

Though carbon-based cathode materials have been most commonly used thus far, metal-based ones have been shown to have promise in improving MESC performance. Carbon materials are advantageous due to their high chemical stability, high bio-compatibility, and relatively high electrical conductivity. Metals, though costly and some not being biocompatible, are advantageous due to their increased mechanical stability, superior electrical conductivity, and catalytic properties. Hence, the combined use of both types of cathode materials takes advantage of the properties of each of these materials [15].

Kracke et al. [16] showed that carbon aerogel cathodes coated with NiMo alloys are a good option for use as a cathode for H_2_-mediated electromethanogenesis. The NiMo alloy was deemed to facilitate the in situ delivery of H_2_, which improved CH_4_ production in the MESC. Furthermore, Gomez et al. [17] showed that injecting Ni and Fe salts into an MESC improved the CH_4_ production of the system, reaching a production rate of 0.83 L (L_c_ d)^−1^. This was attributed to the in situ deposition of Ni and Fe on the carbon felt. The presence of these metals was deemed to improve the conductivity of the cathode, thus facilitating the electron transfer mechanism.

Moreover, recent MESC research has shown a promising contribution of transition metal coatings to the conversion of CO_2_ to volatile fatty acids and CH_4_. In addition, Qiu et al. [18] demonstrated that the use of Sn-modified carbon felt improves the yield of acetate by catalyzing the production of formate, which acts as an electron donor for CO_2_ reduction. Kracke et al. [19] further demonstrated that the use of transition metal-based cathodes, specifically CoP, MoS_2_, and NiMo, resulted in notable rates, selectivity, and stability for methane (CH_4_) and acetate production. This was attributed to the hydrogen evolution reaction (HER) catalytic capabilities of the cathodic materials.

Hence, it is hypothesized that introducing transition metal ions into the MESC cathodic compartment, which comprises a carbon felt (CF) cathode, or modifying the CF cathode by electrodeposition of the same metals, can improve the CO_2_ conversion rate by decreasing the cathode overpotential and/or reducing the resistance for electron transfer. Accordingly, in this study, batch microbial activity assays were employed to evaluate the biocompatibility of several transition metal ions (Ni^2+^, Fe^2+^, Cu^2+^, Sn^2+^, MoO_4_^2−^, and Bi^3+^) with a mixed anaerobic microbial population known to be capable of CO_2_ conversion to CH_4_ and acetate. Subsequent to these assays, the influence of two specific transition metals, namely Bi and Sn, on the CO_2_ conversion was investigated during the MESC operation, first as their addition as ions in the catholyte, and secondly as their presence as metal deposits on the carbon felt cathode, in laboratory-scale MESCs with a continuous supply of CO_2_.

## 2. Results and Discussion

### 2.1. Batch H_2_ and Acetate Consumption Tests

While transition metal ions can be hypothesized to improve bioelectrochemical CO_2_ reduction, some metal ions can inhibit microbial activity, i.e., metal catalysts need to be biocompatible. Thus, a series of batch tests were carried out to evaluate the impact of metal ions towards two key microbial populations responsible for CH_4_ production and expected to be present in the cathodic biofilm in the MESC: hydrogenotrophic and acetoclastic archaea. Notably, acetogenic bacteria capable of producing VFAs from CO_2_ were also expected to be present in the biofilm. The batch tests were carried out using dissolved metal cations and molybdate at relatively high concentrations and under non-bioelectrochemical conditions (no applied current). In the subsequent MESC experiments, two transition metal ions that showed a positive impact on CH_4_ production were added to the MESC cathodic liquid.

The microbial formation of CH_4_ from CO_2_ and H_2_ can be achieved through two competing pathways. While detailed knowledge of bioelectrochemical reactions leading to the conversion of CO_2_ to CH_4_ and acetate is lacking, research agrees that the main reactions that occur during CO_2_ bio-electrochemical conversion can be classified as based on direct and indirect electron transfer. Direct electron transfer (DET) refers to the process by which electroactive microorganisms accept electrons directly from the surface of the cathode. The DET pathway is associated with the electron transfer occurring in the outer membrane of the microbes through cytochromes or membrane-bound redox proteins that are in contact with the biocathode [15,20]. The following reduction reaction, known as direct autotrophic methanogenesis, illustrates direct CH_4_ formation at the cathode surface [21,22]:

(1) $${\mathrm{CO}}_{2}+8H^{+}+8e^{-}↔{\mathrm{CH}}_{4}+2H_{2}O$$

**Figure 1:**
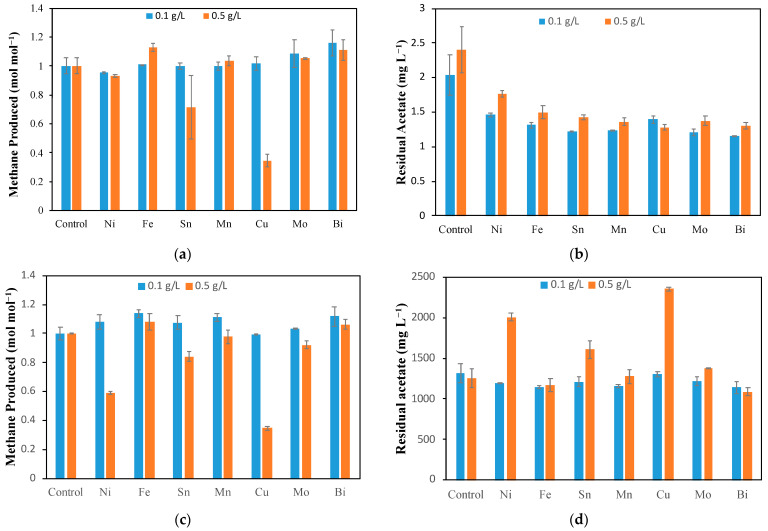
Results of H_2_/CO_2_ consumption test in the presence of metal ions and MoO_4_^2−^ showing (**a**) CH_4_ production and (**b**) residual acetate concentration at the end of the test, and results of acetate batch activity test in the presence of two concentrations of metal ions showing (**c**) CH_4_ production normalized to control and (**d**) residual acetate concentration (mg L^−1^).

Indirect electron transfer corresponds to the mechanism by which CO_2_ conversion products are produced via intermediates, namely hydrogen (H_2_) or acetate, which act as electron donors (Figure 1b). H_2_ can be produced electrochemically on the surface of the cathode via water electrolysis or biotically by some types of electroactive microorganisms in the following reaction occurring in a slightly alkaline environment [23]:

(2) $$2H_{2}O+2e^{-}\;↔H_{2}+2{\mathrm{OH}}^{-}$$

Then, hydrogenotrophic methanogens in the biofilm reduce CO_2_ to CH_4_ via the following bioreaction [21,24]:

(3) $${\mathrm{CO}}_{2}+4H_{2}\to {\mathrm{CH}}_{4}+2H_{2}O$$

Acetate can also be produced directly at the cathode by electroactive microorganisms or indirectly (with H_2_ as an intermediate) by acetogenic microorganisms via the reduction of CO_2_ as follows [25,26,27]:

(4) $$2{\mathrm{CO}}_{2}+4H_{2}\to {\mathrm{CH}}_{3}\mathrm{COOH}+2H_{2}O$$

Finally, acetoclastic methanogens present in the biofilm are able to use the acetate produced to form CH_4_ as follows:

(5) $${\mathrm{CH}}_{3}\mathrm{COOH}\;\to {\;\mathrm{CO}}_{2}+{\mathrm{CH}}_{4}$$

The schematic of these bioelectrochemical transformations is represented in Figure 1b. Accordingly, batch CO_2_/H_2_ consumption tests were conducted to evaluate the impact of transition metals on all three groups of microorganisms.

As mentioned above, in the batch H_2_/CO_2_ consumption tests, dissolved metal salts were added to test bottles to study the impact of these metal ions on the microbial conversion of CO_2_ to CH_4_ and VFAs. Two concentrations of metal ions were used in these tests: 0.1 g L^−1^ and 0.5 g L^−1^. Notably, in addition to Ni^2+^, Fe^2+^, Cu^2+^, Sn^2+^, Mn^2+^, and Bi^3+^ cations, the molybdate (as MoO_4_^2−^) anion was also tested. Figure 1a shows the normalized CH_4_ production (with respect to control), while Figure 1b shows the acetate concentration in the bottles at the completion of the test. Concentrations of other VFAs such as propionate, butyrate, and valerate were below the corresponding detection limits (1 mg L^−1^) and are not shown in the graphs.

For most of metal ions at a concentration of 0.1 g L^−1^, it is evident that there was an insignificant variation in CH_4_ production in the presence of dissolved metal ions and very low residual acetate concentrations, with a slight improvement in CH_4_ production seen in bottles containing Bi^3+^ (16%), MoO_4_^2−^ (8.5%), and Fe^2+^ (1.1%), whereas for 0.5 g L^−1^, the results varied significantly. At the higher concentration of metal ions, inhibition was observed for Sn and Cu ions, with CH_4_ production declining by 15% and 70%, respectively (relative to control). This is validated by various studies that show that Cu is especially toxic to methanogens [28,29,30]. However, at lower Sn and Cu concentrations of 0.1 g L^−1^, there was no negative effect on CH_4_ production.

Other metals showed little to no significant variation in CH_4_ production at both ion concentrations. However, even though the presence of metal ion salts in the bottle tests showed no significant improvement in CH_4_ production, previous studies [16,31] indicated that the use of these metals in an MESC may improve CH_4_ and/or acetate production, as mentioned in the introduction. Such improvement can be either achieved through the improved electrochemical production of H_2_ or by enhancing the DET pathway of CH_4_ production through electromethanogenesis [32]. It is important to note that the impact of metal ions in the bottle tests provides some insight on the toxicity of the metal ions. However, any improved conversion may not translate to the same effects in an MESC. In an MESC, there is an electric current that reduces the metal ions to zero-charge metals that deposit on the cathode surface (electrodeposition). Hence, the effects observed in an MESC to be discussed later in the text are due to a combination of influence of metal ions and metal deposited on the surface of the cathode.

Furthermore, the analysis of acetate concentrations at the end of each test showed some differences (Figure 1b). Although all acetate concentrations at the end of each test were always low (below 3 mg L^−1^) and close to the analytical threshold of 1 mg L^−1^, for all bottles with metal ions, residual acetate concentrations were lower as compared to the control, with a slight deviation among tests containing different metal ions. It is important to note that acetate is simultaneously produced by acetogens and utilized by acetoclastic methanogens as described by Equations (4) and (5). Furthermore, the difference in the remaining acetate concentration is less than 1 mg L^−1^ between control and metal ion bottles (Figure 1b). This amount is very low compared to what is seen in the anaerobic reactors producing biogas. Notably, the low residual concentration of acetate and other VFAs in the CO_2_/H_2_ bottle tests could be indicative of a low acetogenic activity (i.e., insignificant acetate production) as well as high rate of acetate conversion to CH_4_ by acetoclastic methanogens. Considering that the anaerobic sludge used for inoculating the bottles originated from an anaerobic digester that treats agricultural wastes and contained a substantial population of acetoclastic methanogens, it was important to also estimate the impact of metal ions on acetoclastic methanogenesis.

An analysis of H_2_, CO_2_, and CH_4_ headspace concentrations in the test bottles demonstrated a close-to-stoichiometric CH_4_ production (Table 1). Based on Equation (3), every mole of CH_4_ produced needed 4 moles of H_2_ to be used. Indeed, the H_2_-to-CH_4_ ratio was close to 4 for all the tests performed, with the exception of bottles with Cu (at metal ion concentration of 0.5 mg L^−1^), where the value was higher. It can be hypothesized that because of Cu toxicity, products other than CH_4_ and VFAs (e.g., formate) were formed in this case or the biotransformation pathways were affected due to Cu toxicity.

The second part of activity tests was dedicated to evaluating the impact of transition metal ions on methanogenic acetoclastic activity, resulting in the conversion of acetate to CH_4_ and CO_2_ (Equation (5)). The normalized CH_4_ production (with respect to control) observed in these tests is shown in Figure 1c. It is evident that at the low metal ion concentration (0.1 g L^−1^), there was little to no effect on the CH_4_ produced for all metal ions; however, at the concentration of 0.5 g L^−1^, CH_4_ production inhibition was clearly observed for Ni, Cu, and Sn. Ni inhibition led to about a 40% lower CH_4_ production relative to the control, whereas it was about 60% and 20% for Cu and Sn, respectively. When these results were compared with the H_2_ activity test (Figure 1a), it can be concluded that dissolved Cu inhibited both acetoclastic and hydrogenotrophic methanogenic activities. Fe and Bi consistently showed a slight improvement in CH_4_ production at both concentrations, while Mo and Mn showed an improvement only at 0.1 g L^−1^.

Figure 1d shows the residual acetate in the bottles after the end of the experiment. At 0.1 g L^−1^, comparable amounts of acetate were used for all the metal ions and control. However, at 0.5 g L^−1^, we see more residual acetate for Ni, Sn, and Cu. Table S1 (in Supplementary Materials) shows the amount of acetate used, the amount of CH_4_ produced, and the ratio of CH_4_ produced to acetate used. This ratio was about one for all the bottles at both concentrations, which is consistent with the expected reaction stoichiometry (Equation (5)), indicating that although Cu, Ni, and Sn reduced the rate of acetate conversion to CH_4_, the conversion stoichiometry of this microbial biotransformation was not affected. This also suggests that in the biotransformation of acetate, only CH_4_ is predominantly formed even though there were other microbial species present other than acetoclastic methanogen.

From these results, it can be concluded that the presence of transition metal ions had little to no effect on the observed stoichiometry of CH_4_ production from acetate. At the same time, acetate consumption was significantly slower in the presence of 0.5 g L^−1^ of Ni and Cu, i.e., these metals significantly suppressed acetoclastic methanogenic activity. Although there was a 20% inhibition of acetate consumption in the presence of Sn at a concentration of 0.5 g L^−1^, this value was lower than both Cu and Ni inhibition. Furthermore, inhibition was not observed at the lower Sn concentration of 0.1 g L^−1^.

Overall, the batch tests helped to exclude Ni and Cu from further testing in the MESC experiments. Notably, Ni is a well-known HER catalyst [33], while several recent works demonstrated promising catalytic properties of Cu for the direct electrochemical reduction of CO_2_ [7,34,35]. While the utilization of these transition metals at an MESC cathode would support the electrocatalytic conversion of CO_2_, which could be complementary to bioelectrochemical conversion, the high toxicity of these metals towards methanogenic populations would limit microbial activity. Our previous studies with an MESC cathode enhanced by alloy electrodeposition (Ni-Fe and Ni-Fe-Mn alloys) [17,36] showed an improved production of CH_4_ and acetate when using a Ni-based alloy. It can be suggested that the impact of electrodeposited (solid) Ni on CH_4_ production differs from that of Ni cations (Ni^2+^).

Of the remaining three transition metals tested in the batch tests described above, Bi and Sn were of interest due to their known catalytic properties in the electrochemical conversion of CO_2_ [37,38]. Bi performed well and seemed to have a positive contribution to CH_4_ production in both H_2_/CO_2_ and acetate consumption tests. Also, Sn showed no inhibition of CH_4_ production at 0.1 g L^−1^. Moreover, previous research conducted by Qui et al. [18] suggests that Sn facilitated acetate production via the production of formate, which effectively acts as an electron donor. Based on these considerations, it was decided to test the impact of Bi and Sn on CO_2_ conversion in an MESC.

### 2.2. MESC Operation with Bismuth Ion Injection

Following batch tests, the impact of Bi and Sn ions on the bioelectrochemical conversion of CO_2_ to CH_4_ was studied in MESCs with a continuous supply of CO_2_. These cells were inoculated with the same anaerobic sludge as used for batch activity tests, after homogenizing it. Before inoculation, each MESC was operated for 2–5 days under abiotic conditions in order to electrochemically characterize the system and determine the amount of H_2_ gas produced electrochemically.

**Figure 2:**
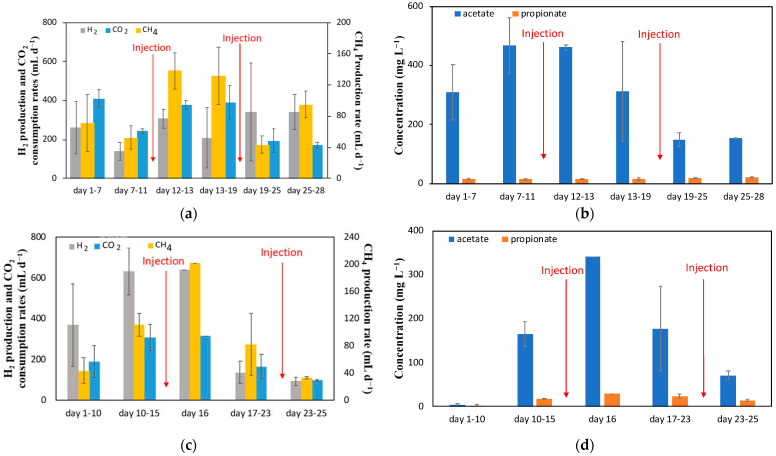
(**a**) H_2_ and CH_4_ production and CO_2_ consumption rates (mL d^−1^), (**b**) VFA concentrations (mg L^−1^) measured during the MESC operation with two Bi ion injections. (**c**) H_2_ and CH_4_ production and CO_2_ consumption rates (mL d^−1^), and (**d**) VFA concentrations (mg L^−1^) measured during the MESC operation with two Sn ion injections.

The results of the Bi injection experiment are summarized in Figure 2a,b, which shows average volumetric rates of product (CH_4_, H_2_, acetate, and propionate) formation as well as CO_2_ consumption. The MESC operation was started at a constant current of 50 mA. Although not shown, at this current and under abiotic conditions, a H_2_ production of around 500 mL d^−1^ was observed, corresponding to an energy consumption of 7 Wh/L_H2_.

Starting from inoculation with anaerobic sludge, the Bi MESC was given 11 days for the biofilm to grow and for CH_4_ production to reach a steady state before introducing the Bi solution. The CH_4_ formation rate before injections was 53 ± 15 mL d^−1^. Bi solution was injected on day 12 to obtain an initial concentration of Bi ions of 0.1 g L^−1^. Following this injection, the CH_4_ production rate increased almost immediately to 138 ± 23 mL d^−1^. Limitations in the supply of CO_2_, and hence the availability of dissolved CO_2_/carbonate, indicated by the low cathode off-gas concentration of CO_2_ (2.4%), was subsequently observed. In order to avoid a CO_2_-limited production of CH_4_, its inflow was increased by 50% to 720 mL d^−1^ on day 16, leading to a CO_2_ off-gas concentration of 62%. It is important to note that the increase in CO_2_ supply was simultaneous with changes in current. Not only could this have affected the microbial population, but an increase in current could cause an increase in H_2_ supplied electrochemically. With an increased CO_2_ supply and simultaneous increase in current, there was an immediate increase in H_2_ consumed, shown in Figure 2a. This indicates that H_2_ was used by either hydrogenotrophic methanogens or acetogens. To avoid H_2_ limitation, the current setpoint was gradually increased from 50 mA to 80 mA, starting on day 15 and ending on day 18.

As expected, the increase in current resulted in an increase in H_2_ gas produced. However, the current increase seems to have negatively affected the methanogenic activity, as seen by the increased standard deviation (higher fluctuations) in CH_4_ production witnessed between days 13 and 19 (Figure 2a). It can be suggested that the increased partial pressure of H_2_ negatively affected acetoclastic methanogens, decreasing the acetate conversion to CH_4_. Furthermore, the increased production of H_2_ could disrupt the biofilm formed on the cathode (carbon felt) surface. Notably, acetoclastic methanogens have a slow growth rate [39] and perform better in biofilm-based systems. A higher growth rate of acetogenic bacteria is expected to promote the proliferation of this microbial population in the biofilm as well as in the cathodic liquid at high dissolved H_2_ concentrations.

Acetate production was observed throughout the entire test with a much lower concentration of propionate also detected (Figure 2b), while the production of other VFAs was negligible. At the MESC startup, some abiotic (electrochemical) acetate production was observed with a steady-state acetate concentration of 35 mg L^−1^ (not shown in the figure). After inoculation, the acetate concentration increased due to acetogenic activity, reaching a steady-state value of about 468 ± 94 mg L^−1^ by day 11 (Figure 2b). Immediately after the first injection of Bi ions, the acetate concentration was maintained at around 461 ± 8 mg L^−1^, before falling to a final measured concentration of 150 mg L^−1^ on day 19.

These changes in acetate concentration over time can be either attributed to a short-term increase in activity of acetoclastic methanogens, which convert acetate to CH_4_, as shown in the batch activity tests, or to a decrease in acetogenic activity following Bi injection. Once the MESC current was increased to 80 mA, acetate concentration decreased likely due to a combination of lower acetogenic activity and the competition for H_2_ between acetogenic bacteria and hydrogenotrophic methanogens as well as the possible adverse impact of the increased partial pressure of H_2_, which could also result in a lower acetate concentration. Furthermore, the formation of products other than CH_4_ and short chain fatty acids cannot be excluded.

The second Bi injection was carried out on day 18. There was a notable decrease in product formation observed immediately after this injection, with CH_4_ production reaching 74 ± 11 mL d^−1^ initially (Figure 2a, days 19–25) before plateauing at 95 ± 17 mL d^−1^ (Figure 2a, days 25–28). Furthermore, the acetate concentration declined towards the end of the test, plateauing at a final concentration of 155 ± 1 mg L^−1^ (Figure 2b).

Before the first Bi injection, the Coulombic efficiency (calculated based on H_2,_ CH_4_, and acetate production) was at 92 ± 20%. After the first injection, the Coulombic efficiency was fluctuating, exceeding 100% initially due to the transition to a new steady state, before converging to about 90%. After the second injection, the Coulombic efficiency stabilized at about 60%. The decrease in Coulombic efficiency, which evidences a decrease in the MESC performance after the second Bi injection, supports the observed reduction in CH_4_ and acetate production rates. This is suggestive of the reduced uptake of electrons by the microbial community particularly for the bioconversion of CO_2_ to CH_4_ and acetate as the Coulombic efficiency determination is based on H_2_ and these two major products of CO_2_ conversion. Thus, a decrease in Coulombic efficiency values suggests the formation of products that are not measured. In particular, the formation of ethanol, medium chain fatty acids such as caproate, and alcohols such as butanol and hexanol was previously observed [40,41].

### 2.3. MESC Operation with Tin Ion Injection

Based on the results of the Bi injection experiment, the MESC with Sn injections was maintained at 80 mA and 720 mL d^−1^ of CO_2_ from the beginning of the experiment. Inoculation was carried out on day zero. Immediately after inoculation, an increase in CH_4_ production was observed, whereas an increase in acetate concentration was observed only after a few days, indicating the development of the biofilm, which can be seen in Figure 2c and Figure 2d, respectively. The steady-state CH_4_ production before Sn^2+^ injection was about 111 ± 17 mL d^−1^, a value almost double that observed with the Bi ion injection experiment. This is expected as starting at a higher current resulted in the availability of more H_2_ gas for product formation.

The first injection was performed on day 15 while the second was performed on day 23. The first injection led to a short-term increase in CH_4_ production (day 16, Figure 2c), which eventually decreased to a much lower level. The first day after injection, 202 mL d^−1^ of CH_4_ was observed, a value about 82% higher than the steady-state production before injection. This value eventually decreased to about 35 mL d^−1^ before the second injection, shown in Figure 2c.

After the second injection, there was no change in CH_4_ production (days 23–25 in Figure 2c). This lack of response of CH_4_ production could be due to the depletion of H_2_ gas in the system, which could be caused by the lack of cathode surface area available for the abiotic electrochemical production of H_2_ by water splitting. Another possibility is that all the H_2_ produced is being consumed to form products that are not measured, as mentioned above.

This hypothesis is supported by Coulombic efficiency estimations showing significantly lower values towards the end of this test. In fact, before any Sn injection, the Coulombic efficiency was about 90% (comparable to that of Bi). After the first injection, the value was stable at close to 100%, indicating that all products were accounted for. After the second injection, the Coulombic efficiency declined to about 22 ± 4%. This trend was the same as that observed with the Bi injections, indicating that the second metal ion injection caused product formation that was not measured.

From Figure 2d, with the first injection, it is evident that acetate levels increased to 341 mg L^−1^ before following a decreasing trend, indicating that the injection of Sn caused a temporary increase in acetogenic activity. Acetate levels eventually decreased to 177 ± 96 mg L^−1^ between days 17 and 23. Unlike the first injection, there was no short-term increase in acetate concentration after the second injection; however, there was a decrease to 71 ± 10 mg L^−1^.

Looking at the results of both metal ion injections, it seems that the injection of metal ions only caused a short-term improvement in CH_4_ production. This aligns with what was seen in the H_2_/CO_2_ activity tests (Figure 1a,b), in which CO_2_ and H_2_ consumption was observed within 24 h. The injection of the metal salts ultimately leads to electrodeposition of the metals on the surface of the cathode, which is negatively charged. Both Sn and Bi are known to help facilitate H_2_ evolution [42,43].

Faraday’s equation indicated that metal ions are immediately deposited on the surface of the cathode. In fact, for 0.1 g L^−1^, it would take Sn only 7 min to completely deposit, whereas it would take only 6 min for Bi, assuming that all current is used for the electrodeposition. Although the actual rate of electrodeposition was slower due to bioelectrochemical and electrochemical reactions occurring simultaneously, the expected concentration of metal ions is expected to diminish quickly, which agrees well with the observed short-term impact of each injection on microbial activity. Therefore, it would be of interest to try including these salts in the nutrient solution (catholyte) or to carry out the electrodeposition of metals before the start-up of MESC operation.

Considering that the electrodeposition of transition metals such as Bi and Sn is expected to improve the electrochemical properties of the carbon felt cathode, in the following tests, the carbon felt cathode was first modified by electrodeposition of the two metals on its surface prior to the CO_2_ conversion experiments in the MESC, and their performance on the CO_2_ conversion to CH_4_ and VFAs was evaluated subsequently.

### 2.4. MESC Operation with Sn- and Bi-Modified Carbon Felt Cathode

**Figure 3:**
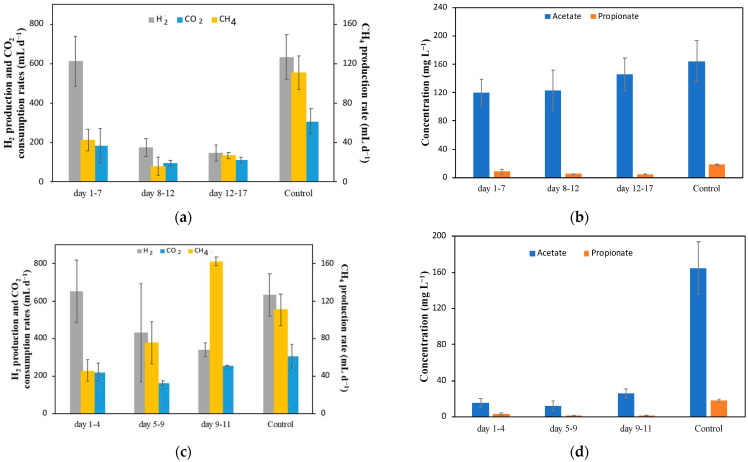
(**a**) Measured off-gas flow rates (mL d^−1^) and (**b**) VFA concentrations (mg L^−1^) in MESC with a Bi-modified carbon felt electrode. (**c**) Measured off-gas flow rates (mL d^−1^) and (**d**) VFA concentrations (mg L^−1^) in an MESC with a Sn-modified carbon felt electrode.

The next part of this research led to the investigation of whether the same two metals (Sn and Bi) would lead to a long-term amelioration of CH_4_ production if they were electrochemically deposited on the surface of the carbon felt before inoculation. It is important to note that the control data that will be used as the basis for comparison in the experiments described further in the text, are the values collected before the injection of metal ions in the previous experiment performed under the same operating conditions (720 mL d^−1^ CO_2_ and 80 mA) (Figure 2c); here, the CH_4_ production averaged at 111 ± 17 mL d^−1^ and that of acetate averaged at 165 ± 29 mg L^−1^ (also shown in Figure 3), corresponding to specific volumetric production rates of CH_4_ and acetate of 555 ± 85 mL (L_c_ d)^−1^ and 66 ± 12 mg (L_c_ d)^−1^, respectively.

**Figure 4:**
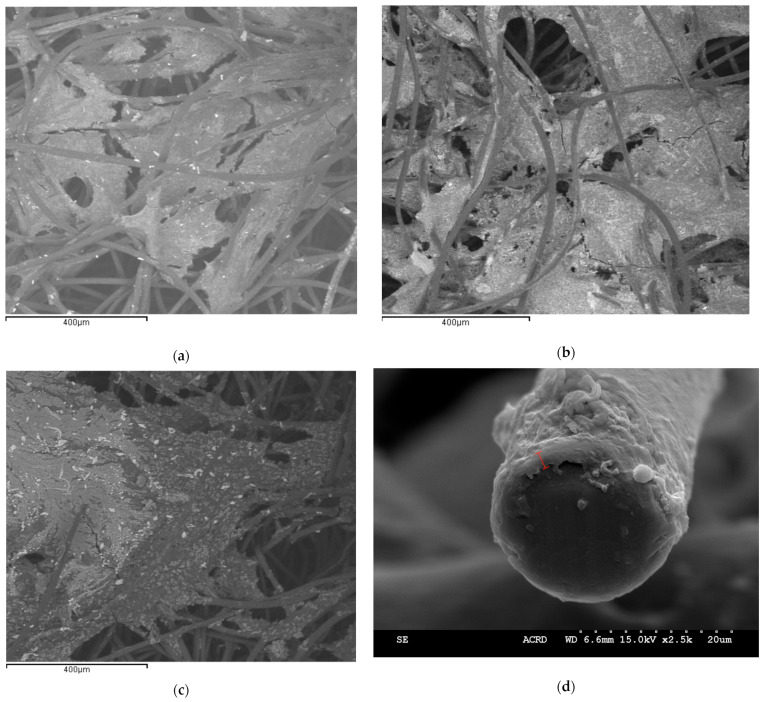
Sample SEM images of the biofilm grown on the surface of a Sn-modified carbon felt (**a**,**b**), and on the (**c**) surface and (**d**) a tip of a strand (with the approximate thickness of the biofilm indicated by a red scalebar) of the Bi-modified carbon felt cathode.

Figure 3 shows the results for the MESC with a Bi-modified carbon felt electrode. The Bi MESC was inoculated on day zero. The electrodeposition of Bi on the carbon felt electrode showed a negative effect in comparison to the control, with a lower level of CH_4_ production as in the Bi injection test, albeit with some fluctuations, as seen in Figure 4a. CH_4_ production in the first few days was high but, eventually, this level lowered by about half (Figure 3a). This could be attributed to the decrease in H_2_ gas available for CH_4_ production. This, in turn, could be due to the biofilm growth which decreases the available surface for the electrochemical formation of H_2_, as discussed previously. However, it could also more likely be because other microbial species have developed within the biofilm, resulting in more competition for H_2_ to be used for the formation of other products. In fact, Coulombic efficiency values in this experiment started at values near 100%; however, this decreased with time, reaching 28.5 ± 6% on average. Again, as with previous experiments, we can hypothesize the production of components that were not measured.

At steady-state, the CH_4_ production rate eventually leveled to about 27 ± 3 mL d^−1^, which is about 24% of the CH_4_ production observed in the previous MESC tests prior to the Sn injections (Figure 2c). The acetate concentration, on the other hand, was 145 ± 23 mg L^−1^, at a level comparable to the control. This indicates that the electrodeposition of Bi adversely affected the conversion rate of CO_2_ to CH_4_, or this suggests that Bi^3+^ enhanced the activity of acetogenic and methanogenic populations, while, on the other hand, Bi metal inhibits methanogenesis (contrary to the observation of the batch activity tests) and has a negligible impact on acetogenesis.

SEM/EDX results (shown later in the manuscript) confirmed the successful electrodeposition of Bi, although it was present at less than 0.05 wt%. The lack of improvement in long-term CH_4_ production may be in part due to the low amount of Bi deposited at the cathode surface.

The abiotic electrodeposition (pre-deposition) of Sn on the carbon felt cathode was carried out under the same conditions as that of Bi (current of 320 mA for 24 h). Figure 3c,d show the gas flow rates and VFA concentrations, respectively, for the MESC experiment on a Sn-modified carbon felt electrode. After inoculation, a consistent increase in CH_4_ production was observed. In fact, compared to the Bi-modified cathode test (Figure 3a,b), the Sn-modified cathode yielded more CH_4_ with time, reaching the highest value of 163 ± 5 mL d^−1^ (815 mL (L_c_ d)^−1^), as can be seen in Figure 3c. Although this CH_4_ production was lower (by 19%) than the maximum achieved in the Sn injection experiment (Figure 2c day 16), the increased production in this MESC was stable over a few days, indicating improvement and stability over a longer period. Furthermore, this showed about a 47% improvement compared to the control experiments, which indicates that Sn, when electrodeposited on carbon felt prior to inoculation as a metal, improved CH_4_ production.

Compared to CH_4_, the H_2_ production rate behaved in the inverse way. This is to be expected since CO_2_ and H_2_ are used for the production of CH_4_ and acetate. Figure 3d shows the average acetate concentration within the MESC. It is important to note that although there was no significant change with time, the acetate concentration was low, reaching a maximum value of 31 mg L^−1^. It is therefore evident that the presence of metallic Sn on the carbon felt surface promotes CH_4_ production by activating the pathway of acetoclastic methanogens (Equation (5)). This aligns with the results seen with the Sn salt injections (Figure 2c).

Coulombic efficiency values were more consistent in this experiment between the beginning and end with an average value of 99.7 ± 26%. This indicates that in this experiment, almost all products (CH_4_ and acetate) were accounted for.

SEM/EDX results (shown in Figure 4) confirmed the electrodeposition of Sn on the carbon felt surface. There was about 1.52 wt% of Sn deposited on the surface. Compared to the Bi-modified carbon felt electrode, the higher CH_4_ production seen in Figure 3c is attributed to the increase in metal presence, which can improve electron transfer. As described above, direct CH_4_ production occurs by direct electron transfer between the cathode and electroactive microorganisms (Equation (1)). The presence of metallic Sn facilitates electron transfer as compared to the electron transfer when just carbon felt is used. As such, the production of CH_4_ without intermediates is facilitated. This process requires less energy and thus improves the efficiency of CO_2_ conversion.

Table 2 below summarizes the results obtained by the experiments employing Sn and Bi both as ions and metals on the cathode. The data show that metal salt injections into an MESC caused a temporary increase in CH_4_ production. Sn and Bi ion injections seem to temporarily improve methanogenic activity, which is consistent to what was seen in the batch activity tests. In all experiments, Sn had more promising results than Bi with higher increases in CH_4_ production, indicating that it is the superior catalyst to Bi for enhancing the bioelectrochemical conversion of CO_2_. The Sn-modified carbon felt electrode especially gave a stable, high CH_4_ production, reaching about 163 ± 5 mL d^−1^ relative to the control. Although this was lower than the CH_4_ production rate observed after the first injection of Sn ions in the MESC experiment (Table 2), when compared to the average of the control values, this shows a clear increase in CH_4_ production.

Interestingly, the results observed in the MESC experiments did not entirely reflect the results obtained in the activity tests. The activity tests showed that Sn reduced the methanogenic activity at a high concentration (0.5 g L^−1^), whereas the presence of Bi improved CH_4_ production both at low and high concentrations. In the MESC experiments, we observed a positive improvement in CH_4_ production when using Sn, both with injected ions and with Sn electrodeposition on the carbon felt cathode before inoculation, whereas Bi had an effect only with ion injections. This observation highlights the difference between the impact of transition metal ions on the microbial (methanogenic and acetogenic) activities and on the catalyst–biofilm interactions in an MESC. While metal ions can disrupt microbial metabolic activity, Sn electrodeposition improved H_2_ production, either through an improved HER or by improving the direct electron transfer to electroactive methanogens and acetogens.

### 2.5. Cathode Characterization

Following the end of each of these experiments, pieces of the cathode were sent for SEM imaging. Figure 4 depicts sample SEM images showing the presence of a biofilm on the Sn- and Bi-modified carbon felt cathode. As can be seen in Figure 4a–c, the biofilm blocked some of the pores of the carbon felt, thus limiting CO_2_, carbonate, and proton transport into the 3D carbon felt cathode, potentially limiting the rate of CO_2_ conversion. Similarly, the diffusion-limited transport of CO_2_ conversion products and H_2_ could be detrimental to the overall rate of CO_2_ conversion, as the accumulation of products and high local pH inside the carbon felt limited bioelectrochemical reactions. This indicates that this could have been a contributing factor to the limited CO_2_ conversion since blocked pores mean that CO_2_/CO_3_^2−^ does not have access to the microorganisms grown within the carbon felt pores. Figure 4d shows a tip of the carbon felt strand of the Bi-modified experiment with grown biofilm. The biofilm thickness could be estimated to be about 24 μm.

The SEM/EDX results confirmed the presence of both Sn and Bi molecules albeit at very low amounts. In the Bi injection experiment, Bi was present at a maximum of 0.55 wt.%, whereas in the Sn injection experiment, there was a maximum of 0.47 wt.% of Sn detected. In the Bi predeposition experiment (Bi-modified carbon felt cathode) (Figure 4c,d), there was less than 0.05 wt.% of Bi on the carbon felt surface, while in the Sn predeposition experiment (Figure 4a,b), Sn was detected at 1.52 wt.%. It is important to note that of all the SEM images, for both injection and electrodeposition, Sn was more consistently measured, whereas in some Bi images, Bi was sometimes not detected on certain parts of the carbon felt surface.

Overall, Sn was present on the surface at a higher amount than Bi, which is to be expected since metallic Sn formation from its salt has a more negative standard electrode potential than that of metallic Bi formation from its salt. Furthermore, Sn salt is more soluble than the Bi salt used for the test. This was the case for both metal injection and predeposition experiments. Importantly, following each injection for metal ion injection experiments, the concentration of Bi and Sn ions declined over time due to the recirculation and washout nature of the MESC.

## 3. Materials and Methods

### 3.1. Batch Tests

Prior to conducting microbial electrosynthesis tests, the conversion of CO_2_ and consumption of acetate in the presence of several transition metals were evaluated in a series of CO_2_ and H_2_ consumption (activity) tests. The test procedure uses sealed serum bottles to incubate anaerobic sludge (inoculum) with a specific carbon source (CO_2_ or acetate).

A phosphate buffer composed of 4 g L^−1^$\;K_{2}{\mathrm{HPO}}_{4}$, 5.1 g L^−1^ ${\mathrm{Na}}_{2}{\mathrm{HPO}}_{4}$, and 1.1 g L^−1^ ${\mathrm{NaH}}_{2}{\mathrm{PO}}_{4}\cdot H_{2}O$ was first prepared. An amount of 3 mL L^−1^ of resazurin 1% solution (*w*/*v*) was added to the phosphate buffer before transferring the solution into a serum bottle and flushing with N_2_/CO_2_ (80/20 vol%) for 30 min. In order to remove the remaining oxygen, the bottle was injected through the rubber stopper with 1 mL of cysteine/Na_2_S solution per 100 mL of solution. All chemicals used for the experiments were analytical-grade.

For the CO_2_ consumption tests, bottles were prepared as follows. Decanted anaerobic sludge was weighed for each bottle to obtain a target concentration of 2 g volatile suspended solids (VSS) per liter. The anaerobic sludge (average 40 g L^−1^ VSS) was obtained from an Upflow Sludge Bed (UASB) reactor treating agricultural wastes at Rougemont, QC, Canada. Duplicate bottles were prepared containing 0 g L^−1^ (control), 0.1 g L^−1^, and 0.5 g L^−1^ of requisite metal salts. The metal salts used were as follows: ${\mathrm{NiSO}}_{4}\cdot 7H_{2}O$, ${\mathrm{FeSO}}_{4}\cdot 6H_{2}O$, ${\mathrm{SnCl}}_{2}\cdot 2H_{2}O$, ${\mathrm{CuSO}}_{4}$, ${\mathrm{Na}}_{2}{\mathrm{MoO}}_{4}\cdot 2H_{2}O$, ${\mathrm{MnSO}}_{4}\cdot 7H_{2}O$, and ${\mathrm{BiCl}}_{3}$. ${\mathrm{Na}}_{2}{\mathrm{MoO}}_{4}\cdot 2H_{2}O$ gives rise to molybdate anions, distinguishing it from the cations formed by the rest of the salts. For the remaining salts, minimal differences were expected from the distinct anions (Cl^−^ and SO_4_^2−^) as both anions were present in the nutrient solution fed to the MESC cathode, as described in the next section.

The phosphate buffer was added to each bottle to obtain a liquid volume of 20 mL in each bottle, which were then flushed with N_2_/CO_2_ (80/20 vol%). The head spaces of the serum bottles were then flushed with H_2_/CO_2_ (80/20 vol%) for 2 min before pressurizing the bottles and incubating them at 35 °C and at 100 rpm using an incubator shaker (Innova 42R, New Brunswick). Gas chromatography was used to analyze the composition of the gas in the headspace: measurements were taken at approximately 30 min, 3 h, 21 h, and 24 h from the start of the experiment.

For the acetate consumption tests, similar steps were followed. Decanted anaerobic sludge was weighed for each bottle to obtain 5 g L^−1^ of volatile suspended solids (VSS). Phosphate buffer and metal salts were added to each bottle, making sure to run them in duplicates. The headspace was flushed with N_2_/CO_2_ (80/20 vol%). A stock solution of sodium acetate was added to each bottle to obtain an initial concentration of 3 g L^−1^. Headspace composition was monitored for two days, taking three measurements daily.

### 3.2. MESC Design and Operation

**Figure 5:**
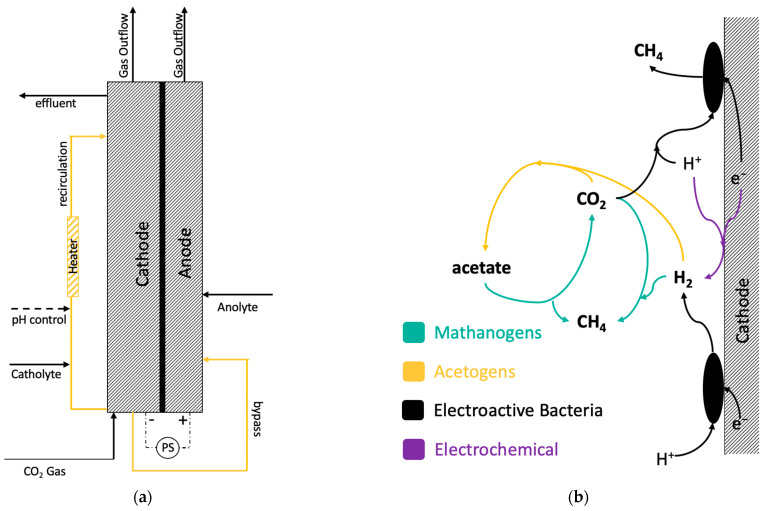
(**a**) MESC schematic with CO_2_ as carbon source, and (**b**) hypothesized pathways of H_2_, CH_4_, and acetate formation at an MESC cathode.

The MESC setup consisted of a two-compartment cell with electrode compartments separated by a dialysis membrane (BioBasic Inc., Markham, ON, Canada) and two layers of nylon cloth, one on each side of the membrane (Figure 5a). The nylon cloth was used as protective layers for the membrane. The volume of the anode compartment was 50 mL, while that of the cathode was 200 mL. The cathode consisted of about 200 mL of densely packed 1 cm × 1 cm × 0.5 cm carbon felt pieces that were connected to the external circuit with a Ti wire. The carbon source was CO_2_ gas which was continuously fed at a rate of 720 mLd^−1^ through a gas sparger installed at the cathode compartment bottom. The cathode compartment was filled with the catholyte composed of 1.34 g L^−1^ $K_{2}{\mathrm{HPO}}_{4}$, 1 g L^−1^ ${\mathrm{KH}}_{2}{\mathrm{PO}}_{4}$, 0.7 g L^−1^ ${\mathrm{NH}}_{4}\mathrm{Cl}$, 3.2 g L^−1^ KCl, 0.5 g L^−1^ yeast extract, and 4 mL L^−1^ of trace metals solution. The composition of the trace metals in the catholyte can be found elsewhere [44].

Two 70 mm × 50 mm × 1 mm titanium iridium oxide meshes were placed in the anodic compartment and connected by Ti wire making up the anode. The anolyte used was a solution of 0.25 M NaOH and the solution used for pH adjustment was 0.125 M H_2_SO_4_. The electrolytes were fed at a rate of 80 mL d^−1^. The temperature at the cathodic compartment was maintained at 25–27 °C by using a flow-through heater in the external recirculation loop and a temperature control system with a temperature probe placed in the cathode compartment. The pH of the catholyte was maintained at 7.5 using a pH controller.

The MESC was operated in constant-current mode, up to 80 mA, and the potential difference was allowed to vary. Furthermore, there was no effluent in the cathode compartment, as all excess cathodic liquid was transferred to the anode compartment through internal connection shown in Figure 5a, i.e., flow-through MESC design was used.

MESC cathodic and anodic off-gas flow rates were measured using a U-tube bubble counter [45]. Off-gas compositions were measured using gas chromatography (HP 6890 GC, Hewlett Packard, Palo Atlo, CA, USA). Gas chromatography (Agilent 6890 N, Santa Clara, CA, USA) was also used to measure the concentrations of volatile fatty acids (VFAs). In addition to gas flow measurements in each compartment, carbon balance calculations were carried out to confirm the observed gas flow rates, taking the amount of carbon fed into the reactor through CO_2_ vs. the amount of carbon present, various products, as well as dissolved CO_2_. Based on this balance, the cathode off-gas flow rate was adjusted such that the inlet and outlet balanced. CO_2_, CH_4_, acetate, and propionate were taken into account. Any other possible products were expected in minimal amounts.

### 3.3. Electrodeposition Tests

MESC experiments were divided into two parts. The first part consisted of two injections of metal salts for in situ electrodeposition during MESC operation. BiCl_3_ and ${\mathrm{SnCl}}_{2}\cdot 2H_{2}O$ were used for these experiments. At the start-up of each experiment, the cathodic compartment was inoculated with 50 mL of homogenized sludge (Lassonde, Rougemont, QC, Canada). The cathodic biofilm was allowed to develop for about 2 weeks of MESC operation, while CO_2_ was continuously fed throughout the cell’s operation. To test the impact of in situ metal deposition on the CO_2_ conversion, 10 mL of a stock solution containing 1 g L^−1^ of either Bi^3+^ or Sn^2+^ ions was injected into the cathode compartment to obtain an initial concentration of 0.05 g L^−1^ of the metal at the test start-up. The MESC was allowed to run for a few days, reducing CO_2_, before a second 10 mL injection of the same metal ion was made. The MESC performance was allowed to stabilize before ending the experiment. The surface of the carbon felt samples were characterized using SEM/EDX.

As a result of the observed low deposition of Sn and Bi in the metal ion injection experiments (shown by EDX results), it was decided to run electrodeposition for the following experiments at a higher current and for a longer time. This was to ensure that metals deposit on the surface and, hence, the impact of deposited and injected metals can be compared. The second part of the MESC tests was dedicated to the electrodeposition of Bi and Sn onto the carbon felt electrode surface before cathode compartment inoculation and CO_2_ conversion. The electrodeposition was achieved using the two-electrode setup shown in Figure 5 with the carbon felt cathode and the titanium iridium oxide meshes as the anode. For Bi electrodeposition, the electrolyte used in the cathode compartment was 0.301 g L^−1^ ${\mathrm{BiCl}}_{3}$, whereas 0.38 g L^−1^ ${\mathrm{SnCl}}_{2}\cdot 2H_{2}O$ was used for Sn electrodeposition. In both solutions, the electrolyte included 20 g L^−1^ $H_{3}{\mathrm{BO}}_{3}$, 25 g L^−1^ NH_4_SO_4_, 35 g L^−1^ NaCl, and 70 g L^−1^ $C_{6}H_{5}{\mathrm{Na}}_{3}O_{7}\cdot 6H_{2}O$. Electrodeposition was carried out at a current of 320 mA during a period of 24 h, followed by the thorough rinsing of the reactor a few times. Subsequently, the surface of the carbon felt samples was characterized using SEM/EDX prior to MESC inoculation and at the end of the experiment.

## 4. Conclusions

In this study, a thorough investigation of seven transition metal ions (Ni^2+^, Fe^2+^, Cu^2+^, Sn^2+^, Mn^2+^, 54^2−^, and Bi^3+^) on hydrogenotrophic and acetoclastic methanogenic activity was carried out. Based on these batch activity tests, it was shown that Ni and Cu ions significantly decreased methanogenic activity, while the presence of Fe and Bi ions improved CH_4_ production from CO_2_ and H_2_. Furthermore, at the relatively low concentration of 0.1 g L^−1^, Sn^2+^ showed no inhibition of methanogenic activity, while it is known to improve the electrochemical production of H_2_. The injection of Bi and Sn ions into the MESC with a continuous CO_2_ supply only demonstrated a temporary increase in CH_4_ and acetate production after the addition of Sn^2+^. The MESC test with the Sn-modified carbon felt cathode resulted in a substantial (up to 54%) increase in CH_4_ production relative to the control (non-modified carbon felt cathode), while the Bi-modified carbon felt cathode showed no improvement in CH_4_ and acetate production. It can be suggested that by optimally selecting transition metals and using metal alloys (e.g., FeSn alloy), both CO_2_ conversion and product specificity can be improved.

One of the most limiting factors in MESCs is electron transfer between the cathode and the microbial biofilm. It can be hypothesized that for cathodes composed of transition metals, which are already known to improve electrochemical H_2_ production, the microbial electrosynthesis of CH_4_ and VFAs from CO_2_ can be improved due to a combination of enhanced electrochemical and bioelectrochemical activities resulting in the direct or indirect (through H_2_) conversion of CO_2_.

## Figures and Tables

**Table 1:** Ratio of H_2_ consumed to CH_4_ produced (mol H_2_/mol CH_4_) observed in the H_2_ activity test in Figure 1a.

Metal Ion	Metal Ion Concentration	
	0.1 g L^−1^	0.5 g L^−1^
Control ^1^	−4.09 ± 0.05	−4.41 ± 0.15
Ni	−4.20 ± 0.03	−4.18 ± 0.01
Fe	−4.23 ± 0.06	−4.12 ± 0.13
Sn	−4.12 ± 0.04	−4.01 ± 0.01
Mn	−4.16 ± 0.03	−4.05 ± 0.04
Cu	−4.19 ± 0.05	−5.76 ± 1.04
Mo ^2^	−4.13 ± 0.03	−4.02 ± 0.1
Bi	−4.13 ± 0.02	−4.01 ± 0.04

^1^ no metal ion addition; ^2^ as MoO_4_^2−^.

**Table 2:** Result summary of MESC operation with Bi and Sn injection or electrodeposition.

	Control ^1^	Metal Ion Injection		Metal Electrodeposition	
		Bi^3+^	Sn^2+^	Bi	Sn
Maximum CH_4_ Production (mL (L_c_ d)^−1^)	640	860	1010	275	835
Maximum acetate concentration (mg (L_c_ d)^−1^)	77.6	222.4	136.4	64.8	12.4

^1^ no metal ion addition.

## Data Availability

Data are available from corresponding author upon request.

## Supplementary Materials

The following supporting information can be downloaded at https://www.mdpi.com/article/10.3390/molecules29020462/s1, Table S1: Stoichiometric ratio of methane produced to acetate used in the acetate activity test at 0.1 g L^−1^ and 0.5 g L^−1^ of the metal ion concentration.
