# Supplementary material for: The Effect of Bismuth and Tin on Methane and Acetate Production in a Microbial Electrosynthesis Cell Fed with Carbon Dioxide
Source: Molecules. 2024 Jan 17;29(2):462. doi: 10.3390/molecules29020462 (PMC10821527; doi:10.3390/molecules29020462)
Supplement: Supplementary file 1 [file molecules-29-00462-s001.zip › molecules-2810851-supplementary.pdf]

The Effect of Bismuth and Tin on the Methane and Acetate Production in a Microbial Electrosynthesis Cell Fed with Carbon Dioxide

Supplementary Material

Table S1. Stoichiometric ratio of methane produced to acetate used in the acetate activity test at 0.1 g L-1 and 0.5 g L-1 of the metal ion concentration.

Metal	Concentration					
	0.1 g L^{-1}			0.5 g L^{-1}		
	Acetate Used (mmol)	Methane Produced (mmol)	Ratio	Acetate Used (mmol)	Methane Produced (mmol)	Ratio
**Control**	0.56	0.54	0.97	0.58	0.57	0.98
**Ni**	0.60	0.59	0.98	0.33	0.34	1.02
**Fe**	0.62	0.62	1.00	0.61	0.62	1.01
**Sn**	0.60	0.58	0.98	0.46	0.48	1.03
**Mn**	0.61	0.59	0.97	0.57	0.56	0.97
**Cu**	0.56	0.54	0.96	0.21	0.20	0.93
**Mo**	0.59	0.56	0.95	0.54	0.53	0.98
**Bi**	0.62	0.61	0.98	0.64	0.61	0.96
